# Biceps femoris long head muscle and aponeurosis geometry in males with and without a history of hamstring strain injury

**DOI:** 10.1111/sms.14619

**Published:** 2024-04-04

**Authors:** Stephanie L. Lazarczuk, Tyler J. Collings, Andrea H. Hams, Ryan G. Timmins, David A. Opar, Suzi Edwards, Anthony J. Shield, Rod S. Barrett, Matthew N. Bourne

**Affiliations:** ^1^ School of Health Sciences and Social Work Griffith University Gold Coast Queensland Australia; ^2^ Griffith Centre of Biomedical and Rehabilitation Engineering (GCORE) Menzies Health Institute Queensland, Griffith University Gold Coast Queensland Australia; ^3^ School of Behavioural and Health Sciences Australian Catholic University Brisbane Queensland Australia; ^4^ Sports Performance, Recovery, Injury and New Technologies (SPRINT) Research Centre Australian Catholic University Melbourne Victoria Australia; ^5^ School of Behavioural and Health Sciences Australian Catholic University Melbourne Victoria Australia; ^6^ Discipline of Exercise and Sport Science, Faculty of Medicine and Health The University of Sydney Camperdown New South Wales Australia; ^7^ School of Environmental and Life Sciences The University of Newcastle Newcastle New South Wales Australia; ^8^ School of Exercise and Nutrition Sciences Queensland University of Technology Brisbane Queensland Australia

**Keywords:** magnetic resonance imaging, muscle‐tendon unit size, tendon, volume

## Abstract

**Objectives:**

Hamstring strain injuries (HSIs) commonly affect the proximal biceps femoris long head (BFlh) musculotendinous junction. Biomechanical modeling suggests narrow proximal BFlh aponeuroses and large muscle‐to‐aponeurosis width ratios increase localized tissue strains and presumably risk of HSI. This study aimed to determine if BFlh muscle and proximal aponeurosis geometry differed between limbs with and without a history of HSI.

**Methods:**

Twenty‐six recreationally active males with (*n* = 13) and without (*n* = 13) a history of unilateral HSI in the last 24 months underwent magnetic resonance imaging of both thighs. BFlh muscle and proximal aponeurosis cross‐sectional areas, length, volume, and interface area between muscle and aponeurosis were extracted. Previously injured limbs were compared to uninjured contralateral and control limbs for discrete variables and ratios, and along the relative length of tissues using statistical parametric mapping.

**Results:**

Previously injured limbs displayed significantly smaller muscle‐to‐aponeurosis volume ratios (*p* = 0.029, Wilcoxon effect size (ES) = 0.43) and larger proximal BFlh aponeurosis volumes (*p* = 0.019, ES = 0.46) than control limbs with no history of HSI. No significant differences were found between previously injured and uninjured contralateral limbs for any outcome measure (*p* = 0.216–1.000, ES = 0.01–0.36).

**Conclusions:**

Aponeurosis geometry differed between limbs with and without a history of HSI. The significantly larger BFlh proximal aponeuroses and smaller muscle‐to‐aponeurosis volume ratios in previously injured limbs could alter the strain experienced in muscle adjacent to the musculotendinous junction during active lengthening. Future research is required to determine if geometric differences influence the risk of re‐injury and whether they can be altered via targeted training.

## 1 INTRODUCTION

Hamstring strain injuries (HSIs) are the most frequent cause of time‐loss from training and competition in running‐based sports.^1^, ^2^, ^3^ Up to 80% of these injuries affect the biceps femoris long head (BFlh), typically at or near its proximal musculotendinous junction (MTJ).^4^, ^5^, ^6^ Reinjuries are common soon after returning to sport^6^, ^7^ and are typically more severe than the initial injury.^7^, ^8^ Several HSI mechanisms have been identified which typically involve a forceful lengthening contraction of the hamstrings.^9^ Most running‐induced HSIs are thought to occur during the terminal swing phase of gait,^10^, ^11^ where the hamstrings actively lengthen and the BFlh experiences peak mechanical strain.^12^, ^13^, ^14^ The magnitude and location of strain experienced at the MTJ is modulated by several factors including the material and geometric properties of the muscle and its aponeurosis. Previously injured BFlh muscles are reported to display chronic atrophy,^15^, ^16^ however, less is known regarding the remodeling of the aponeurosis. As prior injury is considered the most consistent predictor of HSI,^17^, ^18^ understanding changes which occur in both muscle and tendon could be valuable for understanding the mechanisms underpinning high rates of HSI recurrence.

The force generating capacity of a muscle is proportional to its size (number of sarcomeres in parallel)^19^ and it might therefore be expected that a larger muscle would have a relatively larger aponeurosis to effectively transmit forces via the MTJ. It has been proposed that a disproportionately narrower BFlh aponeurosis in comparison to its muscle width may be a risk factor for subsequent BFlh strain injury.^20^, ^21^ Finite element modeling studies and dynamic magnetic resonance imaging (MRI) suggest that a larger BFlh muscle‐to‐proximal aponeurosis width ratio increases the strain experienced during active lengthening by the muscle tissue immediately adjacent to the proximal aponeurosis.^21^, ^22^, ^23^ BFlh aponeurosis size is also reported to be highly variable between individuals and may not be proportional to muscle size,^20^, ^24^ likely leading to variability in the muscle‐to‐aponeurosis ratios between individuals.^20^ However, the only study to examine the relationship between BFlh aponeurosis and muscle size utilized healthy uninjured participants,^20^ and it is unclear whether such relationships might differ in limbs with a history of HSI.

The proximal aponeurosis of BFlh is a complex three‐dimensional tissue. Despite this, prior work has typically limited investigation to two‐dimensional measures of aponeurosis geometry or to measures that do not fully represent its structural complexity.^21^ The authors of these earlier modeling studies acknowledge that the curvilinear measure of width used ignores intramuscular protrusions or “hooks”,^22^ which would subsequently alter the overall aponeurosis size. On a microscopic scale, interdigitations of the MTJ are present which increase the surface area through which force can be transferred.^25^ It is possible that a greater overall interface area, which presumably increases the number of interdigitations present, alters strain experienced in adjacent tissues. Thus, the BFlh interface area, which is a function of the aponeurosis width and through which force is directly transmitted by adjoining tissue, is hypothesized to be important in the distribution of strain.^26^

Prior HSI has been associated with a degree of long‐term MTU remodeling in some but not all studies.^15^, ^16^ For example, Silder et al.^15^ observed atrophy of previously injured BFlh muscles and concomitant hypertrophy of its short head 5–23 months after HSI. Sanfilippo et al.^16^ demonstrated no significant change in BFlh volume 6 months following a return to sport. Further, Freitas et al.^24^ reported that BFlh aponeurosis and muscle volumes were not significantly different between the limbs of professional soccer players with and without a history of HSI. Given moderate‐to‐severe HSI is associated with chronic deficits in BFlh voluntary activation^27^ and altered load sharing,^28^ such a change in stimulus to the hamstrings might result in altered BFlh muscle and/or tendon geometry. Additionally, scar formation following injury may increase aponeurosis size.^16^ Unlike previous studies in healthy populations,^20^, ^26^ no previous studies investigating BFlh MTU geometry in previously injured populations^16^, ^24^ described the ratio between muscle and aponeurosis properties, and analyses were limited to comparisons or correlations between discrete parameters to describe the complex BFlh MTU. While volume provides an overall indication of size of the tissue, it does not thoroughly describe geometric differences along the length of the structure. Given that strain is not evenly distributed along the length of the proximal aponeurosis and that muscle size influences the magnitude of strain experienced,^21^, ^22^ regional differences in the muscle‐to‐aponeurosis size ratio may theoretically alter strain profiles locally. It is also unknown whether region‐specific differences in BFlh muscle and aponeurosis geometry exist in limbs with a history of HSI compared to healthy limbs. Therefore, continuous analyses (i.e., statistical parametric mapping; SPM) may be useful in determining whether and where regional differences exist between limbs with and without history of HSI.

An improved understanding of the impact of prior HSI on BFlh muscle and aponeurosis geometry may have important implications for strategies targeted at reducing the risk of HSI and reinjury. Therefore, the purpose of this exploratory study was to investigate whether global (whole structure) or regional (along length of tissue) differences in BFlh geometry exist between limbs with a history of recent HSI, compared to uninjured contralateral and healthy control limbs.

## 2 MATERIALS AND METHODS

### 2.1 Participants and study design

This cross‐sectional retrospective study involved both original analyses and secondary analyses of previously collected data ^29^, ^30^, ^31^ from 26 recreationally active or sub‐elite males with (*n* = 13) and without (*n* = 13) a history of unilateral HSI. Six original MRIs were collected from participants with a history of unilateral HSI. Secondary processing occurred on 7 of 10 MRIs from injured participants from Bourne et al.^30^ with the remaining three excluded due to unavailable scans. All nine healthy controls from Akhundov et al.^29^ were utilized for secondary analysis. Four healthy controls were randomly selected from Bourne et al.^31^ which had similar inclusion and exclusion criteria to Akhundov et al. All previously injured participants had sustained at least one unilateral HSI in the previous 24 months and had undergone supervised rehabilitation sufficient to return to their pre‐injury levels of training and competition in running‐based sports. Participants were required to be free from musculoskeletal injury to the lower limbs, have no history of traumatic knee injury, and have no history of motor or neurological disorders. All participants completed a standardized MRI screening questionnaire provided by the imaging facility to ensure that it was safe for them to undergo scanning.

Ethical approval was provided by the Queensland University of Technology Human Research Ethics Committee and the University of Queensland Medical Research Ethics Committee,^30^, ^31^ or the Griffith University Human Research Ethics Committee and University of Newcastle Human Research Ethics Committee.^29^ Written informed consent was provided by all participants prior to data collection.

### 2.2 Data acquisition

The MRI acquisition parameters can be found in Table 1. A 3 Tesla (3 T) MRI scanner was used to acquire images of both thighs for all participants. Participants were positioned supine in the magnet bore with the knees fully extended and hips in neutral. Axial T1‐weighted images were acquired from the iliac crest to distal to the tibial plateau. Participants were advised to avoid strenuous activity in the week prior to MRI acquisition.^30^ The six original MRIs collected for the HSI group used the same acquisition parameters as Akhundov et al.^29^

**TABLE 1:** Magnetic resonance imaging acquisition parameters for the three sources of data, including the number of participants from the healthy control and previous hamstring strain injury groups.

	Bourne et al.^30^	Bourne et al.^31^	Akhundov et al.^29^
Scanner	Siemens 3T TrioTim	Siemens 3T TrioTim	GE Medical Systems Discovery MR750w (3T)
Coil	Spinal	Spinal	Lower limb
Body position	Supine, knees fully extended, hips in neutral	Supine, knees fully extended, hips in neutral	Supine, knees fully extended, hips in neutral
Length	Immediately superior to iliac crest to immediately distal to tibial plateau	Immediately superior to iliac crest to tibial condyles	Immediately superior to iliac crest through full lower limb
Parameters	CPMG spin‐echo pulse sequence (transverse relaxation time = 2000 ms; echo time = 10, 20, 30, 40, 50 and 60 ms; number of excitations = 1	Transverse relaxation time = 750 ms, echo time = 12 ms, field of view 400 mm	Sequence name: spin echo; sequence variant: segmented k‐space; repetition time: 3 ms, echo time = 38 ms
Slice thickness	10 mm	10 mm	3 mm
Interslice gap	10 mm	10 mm	3 mm
Number of participants	Seven previous HSI	Four healthy controls	Six previous HSI, nine healthy controls

### 2.3 Data processing

**FIGURE 1:**
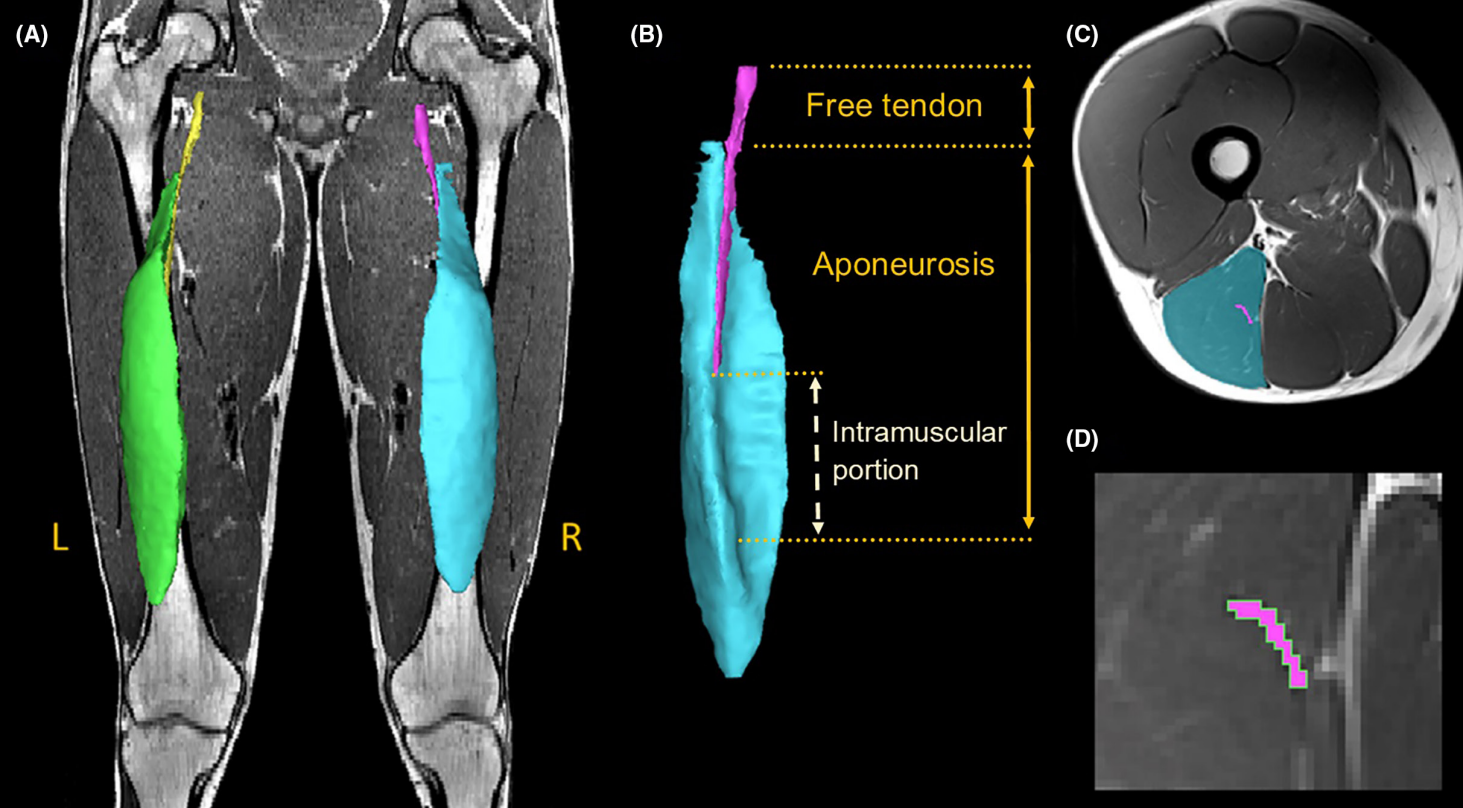
Muscle and tendon segmentation for a representative control participant. (A) Posterior view of a segmentation overlaying MRI, showing muscle tissue for the left (green) and right (blue) biceps femoris long head. Proximal tendon tissue is shown with the left (yellow) and right (purple) free tendon and a small proportion of aponeurosis visible. (B) Anteromedial view of the right BFlh whole MTU displaying muscle and proximal tendon tissue. (C) Axial plane image at approximately 50% of the muscle length of the right thigh with BFlh muscle and aponeurosis colored. (D) Magnified image showing the right BFlh aponeurosis (purple) with spline trace (green) of the interface area between muscle and aponeurosis.

Manual segmentation of deidentified MRIs was conducted in Materialize Interactive Medical Image Control System software (Mimics, v24.0, Materialize) by one author (SLL) who was blinded to group allocation. The boundaries of the following structures were identified and manually segmented on continuous axial slices for both limbs in all participants: BFlh muscle, BFlh proximal aponeurosis, and femurs. Structures were segmented from the most proximal slice in which they were visible, to the most distal slice in which they could be clearly visualized (Figure 1). To determine intra‐rater reliability, the full length of the BFlh muscle and aponeurosis was segmented a second time in an assigned limb from six selected participants (representing 396 CSAs of muscle and 269 CSAs of aponeurosis), with both the limb and participant randomly selected. CSA was used for reliability as other outcome measures were derived from axial traces, i.e., volume, interface area, and ratios. Intra‐rater reliability was calculated using an absolute agreement of a single rater, two‐way mixed effects model (Data S1). Intra‐rater reliability was excellent for muscle CSA (ICC_(3,1)_ = 0.995, 95%CI = 0.991–0.997) and good for aponeurosis CSA (ICC_(3,1)_ = 0.822, 95%CI = 0.779–0.858). The standard error of measurement was 0.2 cm^2^ and 0.01 cm^2^ for muscle and aponeurosis peak anatomical cross‐sectional area (aCSA), respectively. Minimal detectable change was 0.5 and 0.03 cm^2^ for muscle and aponeurosis peak aCSA, respectively.

Interface surface length was determined by manually tracing a line along the contact border between the BFlh muscle and aponeurotic tissue on each axial slice in which it was visible^20^, ^26^ using the “spline” function in Mimics. The length of each spline was calculated as the distance between three‐dimensional co‐ordinates of contact points, and total interface area calculated as the product of summed interface surface lengths and slice thickness.^20^

The CSAs of BFlh muscle and aponeurosis were extracted from axial slices. Muscle and tendon volumes were calculated as the product of the summed axial CSAs and slice thickness. For the purposes of normalization, femur length was established by calculating the distance between the center of the femoral head (fitted with a sphere) to a point 50% the distance between the most prominent points on the medial and lateral femoral epicondyles.

To measure lengths of the MTU structures, three‐dimensional mesh models of structures were created using the “calculate part” tool, followed by the “wrap” tool (smallest detail = 0.71 mm; gap closing distance = 0.36 mm) and “smooth” tool (smoothing factor = 0.4). A centerline was added to BFlh muscle in Mimics and exported to 3‐matic (v16.0, Materialize), along with the aponeurosis mesh. Lengths of MTU structures were determined by measuring the length of the muscle centerline using the “single selection,” and the “distance over the surface” tool for the aponeurosis.

### 2.4 Statistical analysis

A prior finite element modeling study reported substantial differences in BFlh fiber strain with aponeurosis width increases or decreases of 1 standard deviation or *d* = 1.0.^21^ Therefore, a sample size of 13 for each group was deemed sufficient to detect *d* = 1.0 with an alpha of 0.05 and power of 0.7. Statistical analyses were performed using R programming language (version 4.2.1), in RStudio^32^ and Python (version 3.9). Participant descriptive characteristics are presented as mean and standard deviations. Variables were assessed for normality using Shapiro–Wilk tests and visualization of quantile‐quantile plots. Nonparametric tests were deemed most appropriate due to the small sample size, skewed distributions, and presence of outliers. The primary outcome variable of interest was the ratio of muscle‐to‐aponeurosis volume, with muscle and aponeurosis average aCSA, volume, interface area and length considered secondary. The average of left and right limbs was used in the control group as there were no significant differences between these for any outcome variables (all *p* > 0.107). Limbs with a previous HSI were compared with uninjured contralateral limbs using Wilcoxon signed‐rank tests, and to healthy control limbs using Mann–Whitney *U*‐tests. Effect sizes (ES) were calculated using Wilcoxon effect size (*r*) with bootstrapped 95% confidence intervals (95% CI), and interpreted as <0.3 (small effect), 0.30–0.5 (moderate effect) and >0.5 (large effect).^33^

To further investigate the ratio between BFlh muscle and aponeurosis volume, the linear relationship between these variables was determined using Spearman's correlation coefficients (*ρ*). The coefficient of variation (CoV) expressed as a percentage was calculated for all geometric outcome measures for muscle and aponeurosis.

To determine if there were regional differences in BFlh geometry between limbs, BFlh muscle and aponeurosis CSA, interface surface and the muscle CSA‐to‐interface surface length ratio were compared along the length of the tissue using SPM. The “spm1d” library in Python^34^ was used to perform a two‐way ANOVA (group x limb) with limb as a repeated‐measure factor. Prior to conducting the SPM analysis, BFlh muscle and aponeurosis were normalized to 0%–100% of their length using linear interpolation and smoothed by fitting a Local Polynomial Regression line to the data.

The alpha for all statistical testing was set to 0.05.

## 3 RESULTS

### 3.1 Participant characteristics

Twenty‐six recreationally active male adults with (HSI group, *n* = 13, 22 ± 3 years, 182.7 ± 7.1 cm, 83.8 ± 17.3 kg, femur length = 44.7 ± 2.5 cm) and without a history of unilateral HSI (healthy control group, *n* = 13, 22 ± 2 years, 182.5 ± 6.5 cm, 84.2 ± 12.7 kg, femur length = 44.2 ± 2.2 cm) participated in this study. Nine of the 13 previously injured participants had sustained their injury to the BFlh. The remaining four had injuries to either semimembranosus (*n* = 1) or semitendinosus (*n* = 1; not specified/“medial hamstrings” = 2). The rehabilitation period for the HSI participants ranged from 1 to 24 weeks (1–4 weeks, *n* = 6; 6–8 weeks, *n* = 4, 10 weeks, *n* = 1, 24 weeks, *n* = 2). The median time from injury to image acquisition was 5 months (range: 2–24 months). No significant differences were found between groups for age, height, mass, or femur length (all *p* > 0.52).

### 3.2 Muscle and aponeurosis length, average aCSA, and volume

**FIGURE 2:**
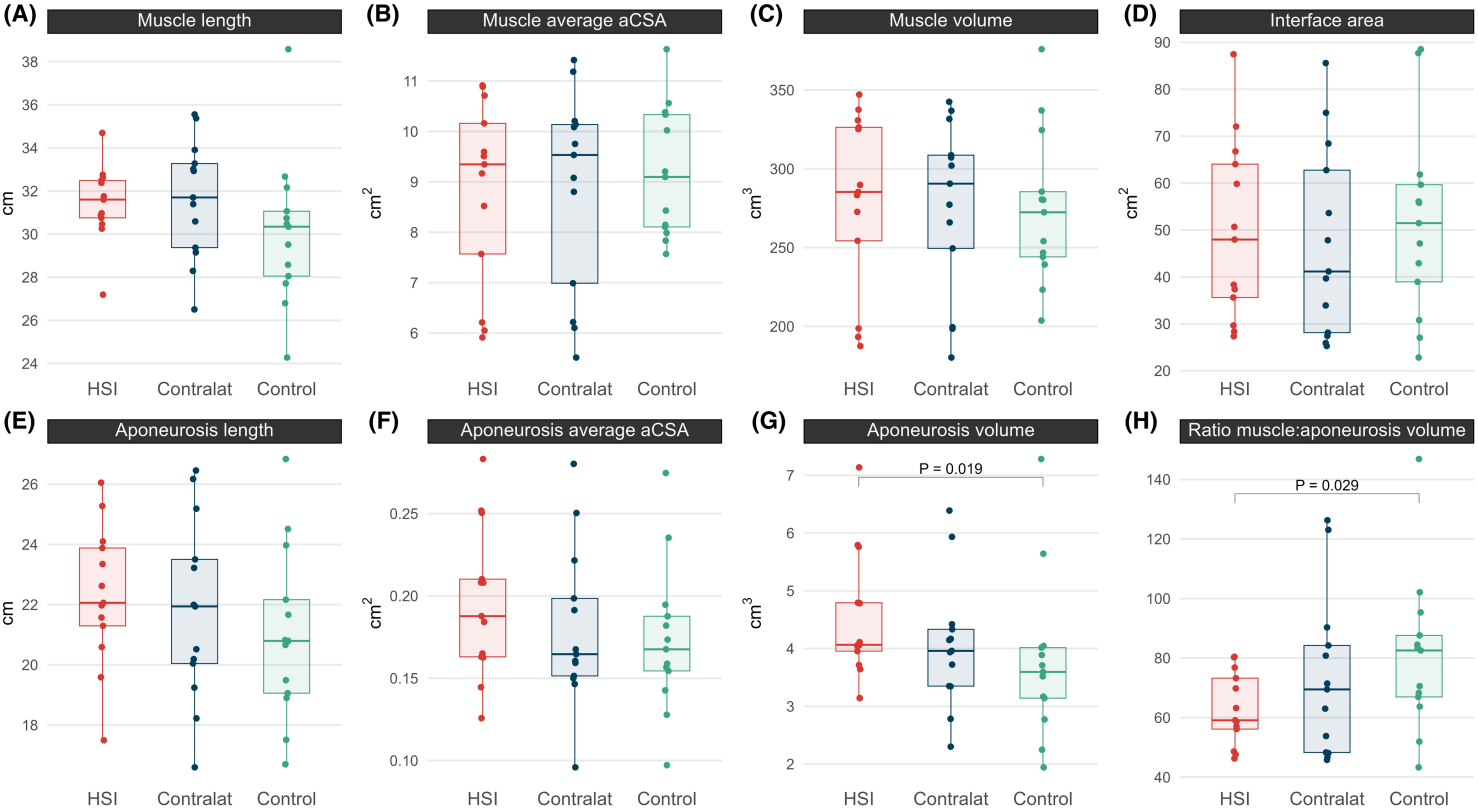
Boxplots of biceps femoris long head muscle length (A), average muscle anatomical cross‐sectional area (aCSA, B), muscle volume (C), proximal aponeurosis interface area (D), aponeurosis length (E), aponeurosis average aCSA (F), aponeurosis volume (G), and muscle‐to‐aponeurosis volume ratio (muscle:aponeurosis, H) for the previously injured (HSI), uninjured contralateral (Contralat) and control limbs. *p* values indicate statistically significant differences between the previously injured and healthy control limbs.

Limbs with previous HSI had significantly greater aponeurosis volumes compared to healthy control limbs (*p* = 0.019, ES = 0.46, 95% CI = 0.12–0.75; Figure 2G). There were no statistically significant differences between limbs with a previous HSI and uninjured contralateral or control limbs for BFlh muscle and proximal aponeurosis length, and average aCSA, interface area, and BFlh muscle volume (*p* = 0.081–1.000, ES = 0.01–0.35; Figure 2A–F). Large variation in BFlh and proximal aponeurosis geometry was observed between participants (muscle CoV: length = 5.7%–11.3%, peak aCSA = 14.2%–21.9%, volume = 17.7%–20.1%; proximal aponeurosis CoV: length = 10.3–13.8%, peak aCSA = 33.3%, volume = 23.9%–36.8%; Data S2).

### 3.3 BFlh muscle‐to‐aponeurosis relationship

**FIGURE 3:**
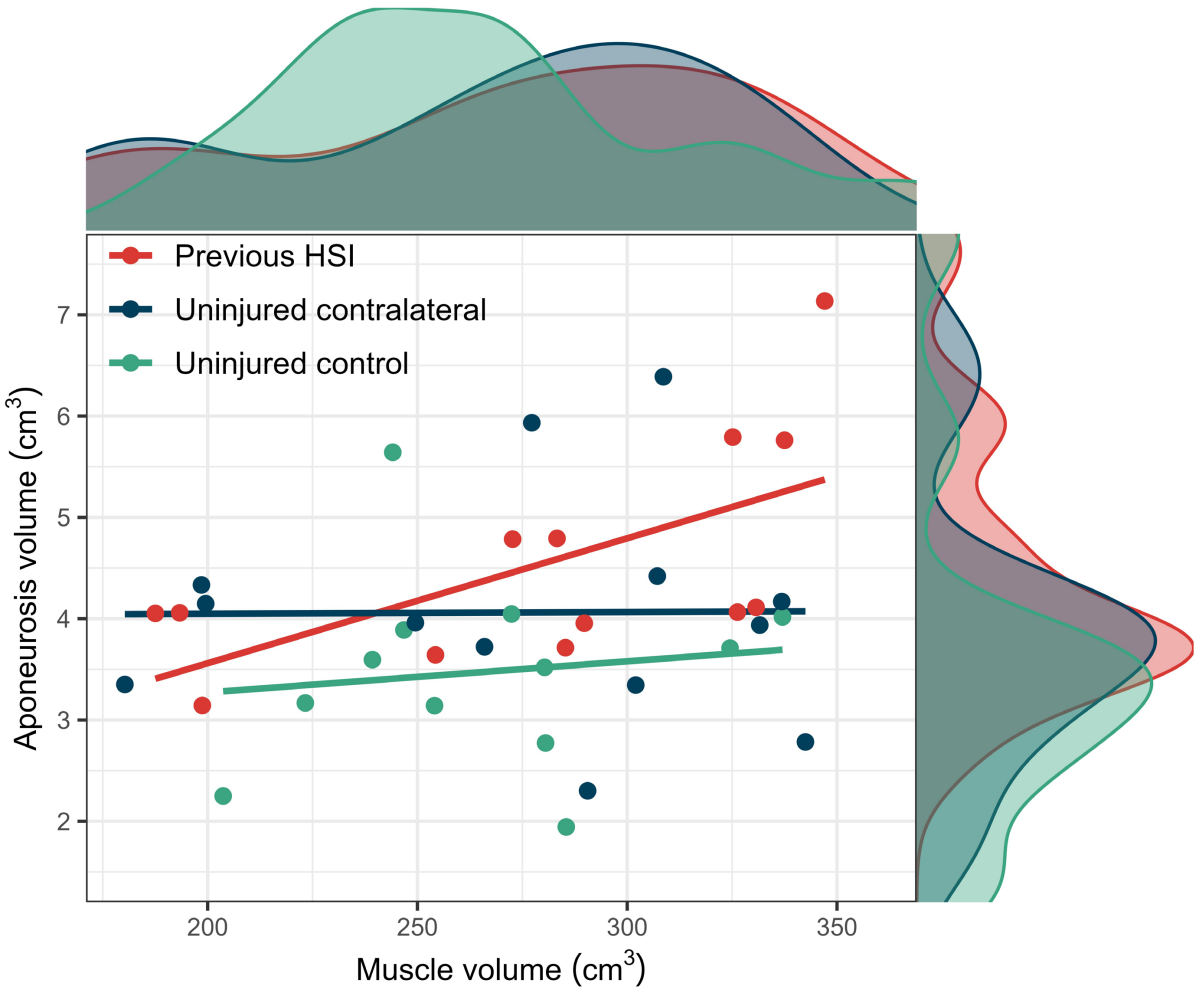
The relationship between biceps femoris long head muscle volume and proximal aponeurosis volume for limbs with a previous hamstring strain injury (HSI), the uninjured contralateral limb and the limbs (averaged) of uninjured controls. Regression lines are shown for each limb. Density plots demonstrate group distribution.

The BFlh muscle‐to‐proximal aponeurosis volume ratio was significantly lower in limbs with a prior HSI compared to the healthy control limbs (*p* = 0.029, ES = 0.43, 95% CI = 0.07–0.73) (Figure 2H, Figure 3). However, the BFlh muscle‐to‐aponeurosis volume ratio was not significantly different between the previously injured and uninjured contralateral limbs of the prior HSI group (*p* = 0.216, ES = 0.36).

There was a significant positive correlation between BFlh aponeurosis and muscle volume in the previously injured limb (*ρ* = 0.64, *p* = 0.022), but not for the uninjured contralateral limb (*ρ* = −0.02, *p* = 0.949), nor healthy control limbs (*ρ* = 0.29, *p* = 0.344; Figure 3).

For all analyses, no differences in results were observed when only including participants who had sustained a BFlh injury (*n* = 9) in comparison to all injured muscles. Additionally, variables were normalized to body mass (aCSA), femur length (length of muscle or aponeurosis), or the product of these (volume, interface area), however, no change in results was seen (Data S3).

### 3.4 Muscle and aponeurosis size along the length of the tissue

**FIGURE 4:**
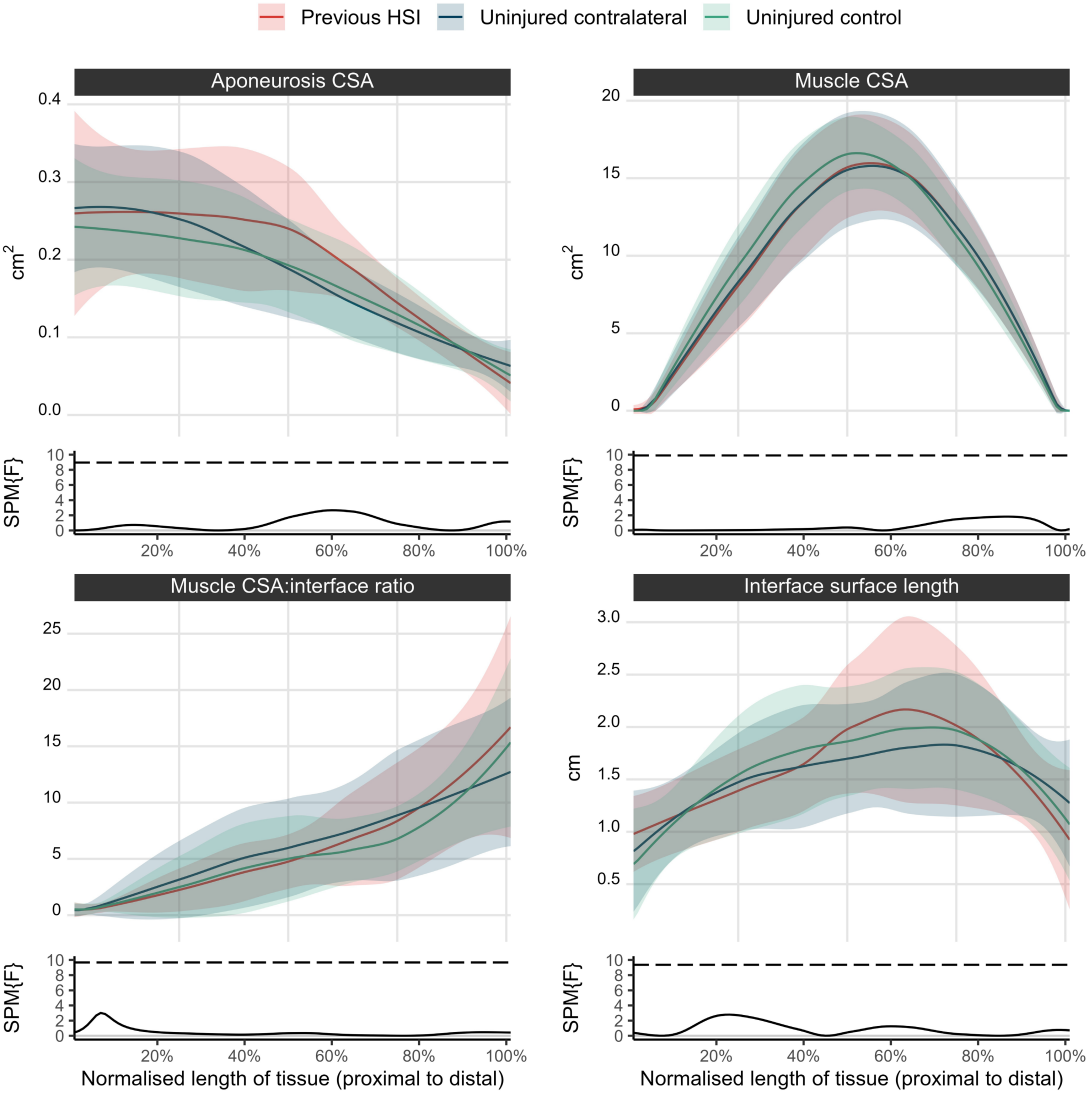
Comparison between legs with a previous hamstring strain injury (HSI), uninjured contralateral leg, and uninjured controls (plotted as average of group legs) for the biceps femoris long head proximal aponeurosis and muscle cross‐sectional area (CSA), interface surface, and the ratio between muscle CSA and interface surface along the length of the tissue. Aponeurosis CSA, the ratio between muscle and aponeurosis, and interface length are relative to the aponeurosis length, while the muscle CSA is relative to the length of the muscle tissue. Groups are compared using statistical parametric mapping (SPM), where the *F* statistic for the interaction between group and leg is indicated by the solid black line, and the critical threshold required for statistical significance (α = 0.05) indicated by the dashed black line.

There were no significant interactions between limb and group for aponeurosis CSA, BFlh CSA, interface surface, or BFlh muscle CSA‐to‐aponeurosis interface area ratio along the relative lengths of the tissue (Figure 4).

## 4 DISCUSSION

This is the first study to investigate both discrete and regional differences in BFlh muscle and aponeurosis geometry between recreational athletes with and without a history of unilateral HSI. The main findings were that: (1) previously injured limbs had significantly larger proximal BFlh aponeuroses which contributed to smaller muscle‐to‐aponeurosis volume ratios compared to healthy control limbs with no history of injury; (2) there were no significant differences in BFlh global or regional muscle geometric properties between previously injured and uninjured contralateral limbs; and (3) no other geometric differences existed between previously injured and healthy control limbs. A smaller BFlh muscle‐to‐aponeurosis volume ratio in previously injured athletes might be expected to alter mechanical strains at or near the proximal musculotendinous junction during forceful eccentric contractions, which may provide more evidence for understanding the mechanism of injury and re‐injury.

Similar to Freitas et al.^24^ we also found no differences in muscle volume and length between limbs with and without a history of HSI. As there were no significant differences in muscle volume between limbs or groups, the smaller muscle‐to‐aponeurosis ratio in the previously injured limb was driven by the significantly larger proximal aponeurosis volumes compared to the control group. A larger BFlh proximal aponeurosis might be expected to reduce the magnitude of strain experienced by the muscle fibers adjacent to the MTJ during forceful eccentric contractions,^21^, ^22^, ^35^ such as the terminal‐swing phase of high‐speed running. Additionally, we found a significant positive correlation between aponeurosis and muscle volume in the previously injured limb, but no correlation in uninjured or healthy limbs. Prior work also reported an absence of correlation in uninjured, healthy limbs, with substantial inter‐individual variation.^20^ The larger aponeurosis volumes in the previously injured limb may be an attempt to provide proportionality between the size of the muscle and aponeurosis. However, the interface area through which force transmission occurs from muscle to tendon did not differ between groups. While it is plausible that the larger aponeurosis volumes in the previously injured limb could confer a protective effect against strain‐induced damage to muscle fibers or the aponeurosis, future studies are needed to determine if these geometric features are prospectively associated with a reduced risk of HSI or re‐injury.

There are several possible interpretations of the primary finding that aponeurosis volume was larger in the previously injured limb. The presence of larger aponeuroses in the previously injured cohort suggests some relationship to injury. However, due to the retrospective nature of the present study, it is not possible to determine whether these preceded or were a direct effect of injury. It is possible that such differences in geometry were present prior to the injury being sustained, although there is currently no prospective evidence examining the effect of aponeurosis geometry on injury risk. In participants who had HSI local to the aponeurosis, larger aponeuroses in previously injured limbs might conceivably result from scar formation at the site of injury.^15^, ^16^ Such thickening of the tendon would increase the CSA locally, and the overall tissue volume globally. However, Sanfilippo et al.^16^ reported no significant BFlh tendon hypertrophy from return to sport to 6 months post‐return to sport, compared to the BFlh tendon in the uninjured contralateral limb. Given the lack of difference between previously injured and uninjured contralateral limbs in the present cohort, post‐injury scar remodeling is an unlikely explanation for the current observations. It is also plausible that the smaller muscle‐to‐aponeurosis volume ratio may be driven by increases in size of both tissues following targeted rehabilitation given that all HSI participants had returned to their pre‐injury activity levels. However, it is not possible to determine the time course of changes in these tissues and therefore their relative plasticity in the current study. A recent systematic review and meta‐analysis demonstrated small increases in size of other lower limb tendons with resistance training,^36^ which is often a key feature of post‐injury rehabilitation and on‐going training for sport. Targeted interventions are known to alter the geometry of the BFlh,^31^, ^37^, ^38^, ^39^ but no study has examined concurrent adaptations in hamstring tendons or aponeuroses. Future studies ought to clarify if BFlh aponeurosis geometry is a potential cause or consequence of HSI, and to determine the effect of training and rehabilitation on the geometry of the whole muscle‐tendon unit.

No differences were found between limbs or groups in relation to muscle‐aponeurosis geometry along the relative length of these tissues. Thus, the increased aponeurosis volume in the previously injured cohort was not a result of regional differences in comparison to other limbs, instead being more likely the summation of small changes along the length of the tissue. Nevertheless, the continuous data results highlight the nonuniformity in muscle CSA, aponeurosis CSA, and interface surface length along the length of the BFlh and reveal that the muscle CSA to interface area ratio is lowest in the most proximal regions of the muscle. These findings might have important implications for future studies aimed at determining the effect of muscle‐tendon geometry on strain experienced by these tissues.

Some limitations of this study should be acknowledged. The retrospective nature of the present study makes it impossible to determine if between‐group differences in BFlh muscle and aponeurosis geometry were present before or after HSI. Although we recruited a relatively homogenous sample of recreationally active or sub‐elite males who participated in running‐based sports, with similar ages and anthropometrics, some factors relating to injury and rehabilitation could not be accounted for. The median time between injury and MRI was 5 months (range = 2–24 months), and it might be argued that participants were at different stages of recovery, despite all having returned to pre‐injury levels of activity. Further, the heterogeneity of severities based on time receiving medical input (1–24 weeks) may alter findings, as a minor strain injury might be expected to have fewer long‐term deficits. The precise site of injury was unknown, and therefore no direct comparisons could be made between the injury site and corresponding contralateral tissues (i.e., at the same level) for any morphological variable. Controlling for time since injury and injury location in future studies may more thoroughly describe post‐injury time course changes in anatomy.^16^ The current study is a secondary analysis of three data sets which used differing MRI acquisition parameters. Although the intra‐rater reliability for all outcome measures was good to excellent (ICC_(3,1)_ = 0.743–0.998), those with thicker axial slices may have limited sensitivity to incremental change along the length of the tissue. Finally, the current study only examined the geometry of the proximal BFlh aponeurosis on the basis that this is where most HSIs occur,^4^, ^5^, ^6^ but future work should consider exploring the geometry of the distal aponeurosis, alongside material properties of muscle‐tendon tissues, which might also influence BFlh strain.^23^

To summarize, aponeurosis geometry differed between limbs with and without a history of HSI. The significantly larger BFlh proximal aponeuroses and smaller muscle‐to‐aponeurosis volume ratios in previously injured limbs in comparison to healthy controls may, theoretically, alter the strain experienced during active lengthening in the tissue immediately adjacent to the MTJ. Additional research is required to understand the implications of larger aponeuroses in hamstring injury and re‐injury risk.

## 5 PERSPECTIVE

This study demonstrated that previously injured BFlh muscles display significantly smaller muscle‐to‐aponeurosis volume ratios and larger proximal BFlh aponeuroses than limbs with no history of injury. These findings might have implications for the risk of HSI or reinjury given evidence that a disproportionately small proximal BFlh aponeurosis relative to the size of its adjoining muscle has been shown to increase mechanical strains in the commonly injured MTJ during active lengthening.^20^, ^21^ Given the interdependence of these tissues, practitioners and researchers should consider the whole muscle‐tendon unit structure rather than one tissue in isolation. Additional work is required to identify if the BFlh muscle‐to‐aponeurosis ratio is associated with the risk of HSI or re‐injury, and whether it can be altered via targeted training.

## FUNDING INFORMATION

Data included from Bourne et al.^30^, ^31^ was supported by funding from the Queensland Academy of Sport's Centre for Sport Performance, Innovation and Knowledge Excellence (SPIKE). Data included from Akhundov et al.^29^ was financially supported by the National Basketball Association and GE HealthCare Orthopedics and Sports Medicine Collaboration.

## CONFLICT OF INTEREST STATEMENT

The authors have no conflicts of interest to declare.

## PATIENT CONSENT STATEMENT

Written informed consent was provided by all participants prior to data collection.

## PERMISSION TO REPRODUCE MATERIAL FROM OTHER SOURCES

Not applicable.

## Supporting information


Data S1:

## Data Availability

Data are available via request to the corresponding author.
