# Supplementary material for: Biceps femoris long head muscle and aponeurosis geometry in males with and without a history of hamstring strain injury
Source: Scand J Med Sci Sports. 2024 Apr 4;34(4):e14619. doi: 10.1111/sms.14619 (PMC12810454; doi:10.1111/sms.14619)

SUPPLEMENTARY INFORMATION FOR:

TITLE: Biceps femoris long head muscle and aponeurosis geometry in males with and without a history of hamstring strain injury

In: Scandinavian Journal of Medicine & Science in Sports

AUTHORS

Lazarczuk, Stephanie L. ^1,2^ (ORCID: 0000-0001-8467-8799)

Collings, Tyler J. ^1,2^ (ORCID: 0000-0002-2050-6442)

Hams, Andrea H. ^1,2^ (ORCID: 0000-0003-2908-4271)

Timmins, Ryan G. ^3,4^ (ORCID: **0000-0003-4964-1848)**

Opar, David A. ^4,5^ (ORCID: 0000-0002-8354-6353)

Edwards, Suzi ^6,7^ (ORCID: 0000-0002-5790-0232)

Shield, Anthony J. ^8^ (ORCID: 0000-0002-0393-2466)

Barrett, Rod S. ^1,2^ (ORCID: 0000-0002-1784-1629)

Bourne, Matthew N. ^1,2^ (ORCID: 0000-0002-3374-4669)

AFFILIATIONS

1. School of Health Sciences and Social Work, Griffith University, Gold Coast, QLD, Australia
2. Griffith Centre of Biomedical and Rehabilitation Engineering (GCORE), Menzies Health Institute Queensland, Griffith University, Gold Coast, QLD, Australia
3. School of Behavioural and Health Sciences, Australian Catholic University, Brisbane, QLD, Australia
4. Sports Performance, Recovery, Injury and New Technologies (SPRINT) Research Centre, Australian Catholic University, Melbourne, VIC, Australia
5. School of Behavioural and Health Sciences, Australian Catholic University, Melbourne, VIC, Australia
6. Discipline of Exercise and Sport Science, Faculty of Medicine and Health, The University of Sydney, Camperdown, NSW, Australia
7. School of Environmental and Life Sciences, The University of Newcastle, Ourimbah, NSW, Australia
8. School of Exercise and Nutrition Sciences, Queensland University of Technology, Brisbane, Australia

CORRESPONDING AUTHOR

Stephanie L. Lazarczuk

School of Health Sciences and Social Work, G02 Clinical Sciences 2

Griffith University, Gold Coast Campus

Parklands Drive, Parklands, QLD, 4222

Australia

+61 422278156

[stephanie.lazarczuk@griffithuni.edu.au](mailto:stephanie.lazarczuk@griffithuni.edu.au)

S1. Intraclass correlation coefficients (ICC) with 95% confidence intervals (95%CI) for axial segmentation of biceps femoris long head (BFlh) muscle and aponeurosis tissue. Values are subdivided by the origin of the MRI (either Bourne et al. (2016, 2017) or Akhundov et al. (2022)), with the number of MRI axial slices (n) included in the reliability analysis.

|  |  | n | ICC | 95%CI lower bound | 95%CI upper bound | Interpretation |
| --- | --- | --- | --- | --- | --- | --- |
| BFlh Muscle | All | 396 | 0.995 | 0.991 | 0.997 | Excellent |
|  | Bourne et al. | 94 | 0.998 | 0.997 | 0.999 | Excellent |
|  | Akhundov et al. | 302 | 0.994 | 0.986 | 0.996 | Excellent |
| BFlh Aponeurosis | All | 269 | 0.822 | 0.779 | 0.858 | Good |
|  | Bourne et al. | 68 | 0.951 | 0.922 | 0.970 | Excellent |
|  | Akhundov et al. | 201 | 0.743 | 0.672 | 0.800 | Moderate to good |

Note: ICC (3,1) = absolute agreement of a single rater, two-way mixed effects model.

References:

Akhundov, R., et al. (2022) Is subject-specific musculoskeletal modelling worth the extra effort or is generic modelling worth the shortcut? *PLoS One*. 17: 1-16.

Bourne, M., et al. (2016) Muscle activation patterns in the Nordic hamstring exercise: Impact of prior strain injury. *Scandinavian Journal of Medicine and Science in Sports*. 26: 666-674.

Bourne, M., et al. (2017) Impact of the Nordic hamstring and hip extension exercises on hamstring architecture and morphology: implications for injury prevention. *British Journal of Sports Medicine*. 51(5):469-77.

S2. Coefficients of variation (CoV) for each morphological variable for the control (CON) and previous hamstring strain injury (HSI) groups.

|  | **CON (n = 13)** |  | **HSI (n = 13)** | |
| --- | --- | --- | --- | --- |
| Variable | CoV (%) | Injured limb  CoV (%) | | Uninjured limb CoV (%) |
| Peak aCSA |  |  | |  |
| Muscle | 14.2 | 19.0 | | 21.9 |
| Normalised muscle | 15.0 | 15.0 | | 19.0 |
| Aponeurosis | 33.3 | 33.3 | | 33.3 |
| Normalised aponeurosis | 18.1 | 27.5 | | 33.3 |
| Muscle:aponeurosis ratio | 26.5 | 18.2 | | 31.5 |
| Volume |  |  | |  |
| Muscle | 17.7 | 20.1 | | 19.9 |
| Normalised muscle | 12.6 | 22.1 | | 19.6 |
| Aponeurosis | 36.8 | 23.9 | | 26.8 |
| Normalised aponeurosis | 20.0 | 25.0 | | 36.4 |
| Muscle:aponeurosis ratio | 31.7 | 19.8 | | 37.5 |
| Length |  |  | |  |
| Muscle | 11.3 | 5.7 | | 9.4 |
| Normalised muscle | 14.3 | 14.3 | | 14.3 |
| Aponeurosis | 13.8 | 10.3 | | 13.8 |
| Normalised aponeurosis | 20.0 | 20.0 | | 20.0 |
| Interface area | 34.9 | 36.5 | | 31.3 |
| Normalised interface area | 39.3 | 29.7 | | 26.1 |
| Muscle volume:interface area ratio | 35.2 | 40.7 | | 33.9 |

S3. Boxplots of normalised biceps femoris long head muscle and proximal aponeurosis length, average anatomical cross-sectional area (aCSA), volume, interface area and muscle-to-aponeurosis volume ratio (muscle:aponeurosis) for the previously injured (HSI), uninjured contralateral (Contralat) and control limbs.


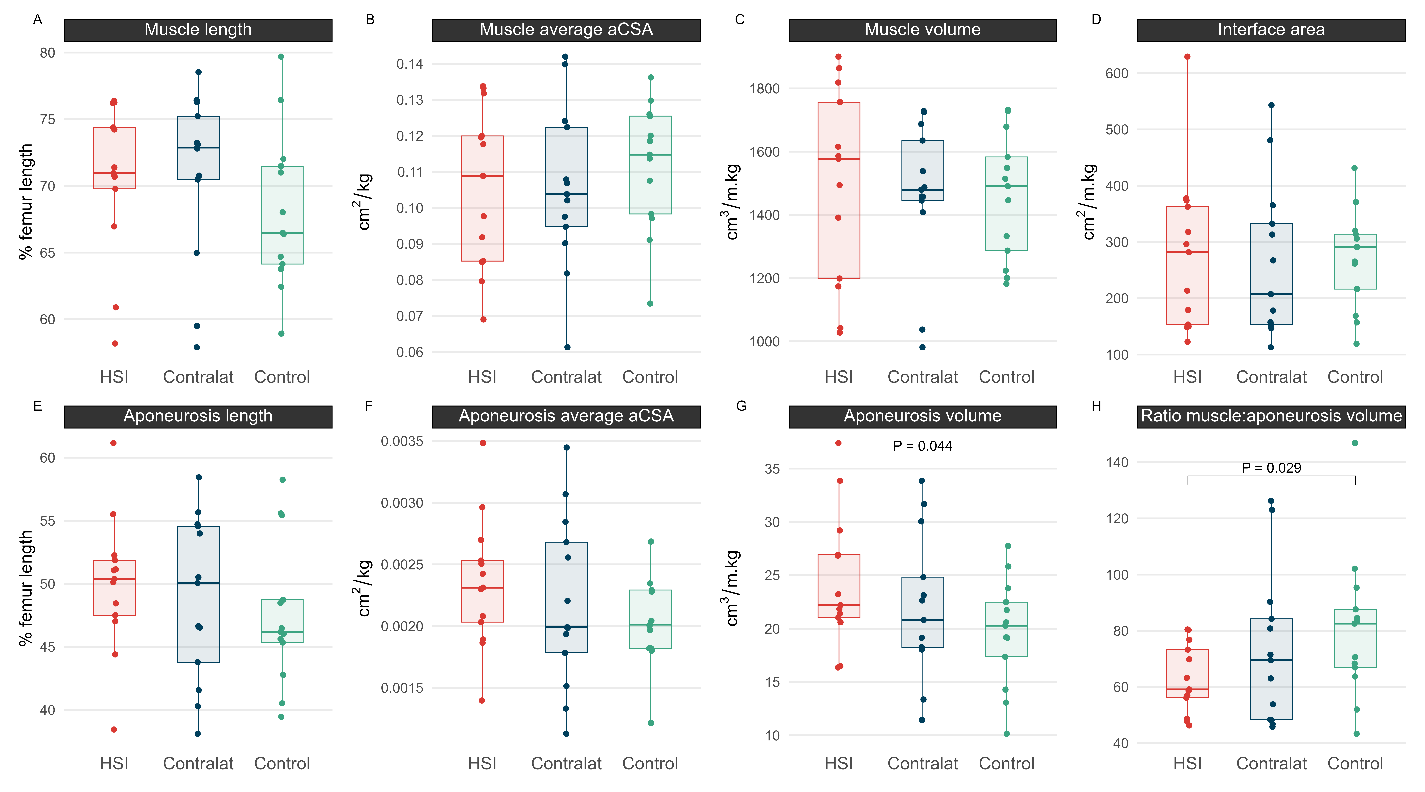

Supplement: Supplementary file 1 — Data S1: [file SMS-34-e14619-s001.docx]
